# Resting Energy Expenditure and Cold-induced Thermogenesis in Patients With Overt Hyperthyroidism

**DOI:** 10.1210/clinem/dgab706

**Published:** 2021-09-27

**Authors:** Claudia I Maushart, Jaël R Senn, Rahel C Loeliger, Judith Siegenthaler, Fabienne Bur, Jonas G W Fischer, Matthias J Betz

**Affiliations:** Department of Endocrinology, Diabetes and Metabolism, University Hospital Basel, University of Basel, Petersgraben 4, CH-4031 Basel, Switzerland

**Keywords:** brown adipose tissue, hyperthyroidism, energy expenditure, cold induced thermogenesis, thyroid hormone

## Abstract

**Context:**

Thyroid hormone (TH) is crucial for the adaptation to cold.

**Objective:**

To evaluate the effect of hyperthyroidism on resting energy expenditure (REE), cold-induced thermogenesis (CIT) and changes in body composition and weight.

**Methods:**

This was a prospective cohort study at the endocrine outpatient clinic of a tertiary referral center. Eighteen patients with overt hyperthyroidism were included. We measured REE during hyperthyroidism, after restoring euthyroid TH levels and after 3 months of normal thyroid function. In 14 of the 18 patients, energy expenditure (EE) was measured before and after a mild cold exposure of 2 hours and CIT was the difference between EEcold and EEwarm. Skin temperatures at 8 positions were recorded during the study visits. Body composition was assessed by dual X-ray absorption.

**Results:**

Free thyroxine (fT4) and free triiodothyronine (fT3) decreased significantly over time (fT4, *P* = .0003; fT3, *P* = .0001). REE corrected for lean body mass (LBM) decreased from 42 ± 6.7 kcal/24 hour/kg LBM in the hyperthyroid to 33 ± 4.4 kcal/24 hour/kg LBM (–21%, *P* < .0001 vs hyperthyroid) in the euthyroid state and 3 months later to 33 ± 5.2 kcal/24 hour/kg LBM (–21%, *P* = .0022 vs hyperthyroid, overall *P* < .0001). fT4 (*P* = .0001) and fT3 (*P* < 0.0001) were predictors of REE. CIT did not change from the hyperthyroid to the euthyroid state (*P* = .96). Hyperthyroidism led to increased skin temperature at warm ambient conditions but did not alter core body temperature, nor skin temperature after cold exposure. Weight regain and body composition were not influenced by REE and CIT during the hyperthyroid state.

**Conclusion:**

CIT is not increased in patients with overt hyperthyroidism.

Hyperthyroidism is a common endocrine disease which, among other symptoms, often leads to weight loss and heat intolerance (1). Thyroid hormones (THs) are pivotal regulators of human energy expenditure (EE) and weight loss is mainly due to an increased resting energy expenditure (REE) (2). The high levels of TH in hyperthyroidism affect virtually all mammalian cells (3). Brown adipose tissue (BAT) contributes to EE and heat production especially in response to mild cold exposure (4, 5).

BAT is thermogenic and can directly convert chemical energy stored in lipids and carbohydrates into heat. The tissue is densely innervated and vascularized. Brown adipocytes contain numerous mitochondria and store lipids in small intracellular droplets which can undergo rapid lipolysis upon stimulation by norepinephrine (5). The resulting fatty acids activate uncoupling protein 1 (UCP1), a unique protein in BAT mitochondria. UCP1 short-circuits the proton gradient across the inner mitochondrial membrane thereby dissipating the stored energy as heat (6). BAT is activated by the sympathetic nervous system in response to mild cold exposure. Its physiological function in humans is to efficiently generate heat in order to maintain a constant core body temperature of approximately 37°C without the need for shivering. The increase in energy expenditure (EE) above baseline levels in response to mild cold exposure is called “cold-induced thermogenesis” (CIT) and is a major function of BAT (5). However, it should be noted that probably also skeletal muscle and other tissues may contribute to CIT (7). During the past decade, BAT and CIT have gained the attention of biomedical research as both may constitute attractive targets to treat obesity and metabolic diseases (8). Early on, it has been recognized that TH is crucial for mitochondriogenesis and adaptation of BAT activity in response to cold exposure (9-11). The tissue expresses high levels of deiodinase 2 (DIO2) which converts thyroxine (T4) to triiodothyronine (T3) (12). Importantly, cold exposure via adrenergic stimulation dramatically increases DIO2 expression in BAT (13). We and others demonstrated previously that CIT and BAT activity are reduced in patients suffering from hypothyroidism and can be restored to normal levels by adequately replacing levothyroxine (14, 15).

The canonical pathway of BAT activation is via the sympathetic nervous system (SNS) and its main transmitter norepinephrine in response to cold exposure (5). Thus a cold stimulus is usually necessary to detect and quantify BAT activity in healthy humans (16). Interestingly, assessment of BAT activity by ^18^F-fluorodeoxyglucose positron emission tomography/computed tomography in patients suffering from hyperthyroidism revealed increased BAT activity even in the absence of cold exposure (17). However, cold-induced BAT activity and CIT in hyperthyroid humans have not been studied yet.

In this prospective cohort study of patients who were treated for hyperthyroidism we therefore aimed to investigate the effect of TH status on CIT. Moreover, we were interested if TH induced changes in energy metabolism corresponded to changes in body weight and composition during the course of the treatment.

## Subjects and Methods

### Subjects

We conducted a prospective observational study in patients with overt hyperthyroidism. Between January 2018 and February 2020 we enrolled 19 patients, age 20-70 years, presenting to the outpatient endocrine clinic at the University Hospital Basel. To meet inclusion criteria, patients had to have a thyroid-stimulating hormone (TSH) value below 0.2 mU/L and a level of free T4 (fT4) at or above 25 pmol/L or free T3(fT3) at or above 8 pmol/L. We excluded patients with a body mass index (BMI) above 30 kg/m^2^, uncontrolled diabetes (glycated hemoglobin [HbA1c] > 7.5%), asthma, chronic obstructive pulmonary disease, or any other significant chronic or acute disease such as heart or kidney failure, liver cirrhosis, or metastasized cancer, pregnancy or breastfeeding, known hypersensitivity to cold, such as primary or secondary Raynaud’s syndrome, or abuse of alcohol or illicit drugs.

The study protocol was reviewed and approved by the medical ethics committee at the University of Basel (ID EKNZ 2017-02044) and registered on ClinicalTrials.gov (NCT03379181). All participants provided written informed consent prior to enrollment to the study.

### Study Flow

Patients were screened within 3 days of the diagnosis of hyperthyroidism whether they were eligible to participate. The study visits took place at 3 time-points during the course of treatment: All participating patients were seen as soon as possible after the screening while still hyperthyroid. The mean time between screening and the first visit was 6.4 ± 2.4 days (range 1-10 days). Antithyroid treatment was started or continued as indicated by the treating physician according to standard of care. It was not influenced by the participation in the study.

Once euthyroid levels off T4 and fT3 were reached patients were invited for the first follow-up visit. After at least 3 months of stable, euthyroid hormone levels the last study visit was conducted.

At each visit we measured clinical parameters, performed thyroid function tests, and assessed REE and body composition. Additionally, 14 patients agreed to participate in assessment of CIT at each visit.

### Clinical Parameters

In all participants, weight and height were measured and BMI was calculated (kg/m^2^). Additionally, blood pressure (mmHg), and pulse rate (bpm) were measured.

### Laboratory Parameters

In all subjects, serum TSH, fT3, fT4, and HbA1c) were measured. All routine analyses were conducted at the central laboratory of the University Hospital Basel except for HbA1c, which was measured at the point of care (DCA Vantage, Siemens Medical Systems, Erlangen, Germany). For TSH and fT3/fT4 electrochemiluminescence immunoassays (Elecsys, all assays from Roche Diagnostics GmbH, Mannheim, Germany) were used. The reference range for TSH was 0.332 to 4.490 mIU/L. The fT3 and fT4 assays had a reference range of 2.6 to 5.6 pmol/L and 11.6 to 22.0 pmol/L, respectively.

### Measurements of Energy Expenditure

All measurements of EE took place in an air-conditioned study room at a controlled ambient temperature of 24°C year round. Subjects fasted during 6 hours prior to the study visit. In addition, they were asked to refrain from intensive physical exercise 24 hours prior to the respective study visit. For determination of EE, participants were placed in a hospital bed and were covered with a fleece blanket. In all participants EE was measured by indirect calorimetry for 30 minutes using a ventilated hood calorimeter (Quark RMR, Cosmed, Rome, Italy).

### Cold-induced Thermogenesis

CIT is defined as the difference between EE during warm conditions (EE_warm_) and after mild cold exposure (EE_cold_). After the measurement of EE_warm_, the blanket was removed, the patients were asked to only wear a t-shirt and shorts. They were exposed to a mild cold stimulus using a water-circulated cooling system (Hilotherm clinic, Hilotherm GmbH, Germany) placed around the patient’s waist. The water temperature was continuously reduced by 1°C every 2 minutes from 25°C to a minimum of 10°C. Participants were periodically asked about their sensation of cold according to a visual analog scale (VAS) and if they noticed shivering. In case of shivering, they were covered with a blanket for 5 minutes and the water temperature was raised by 2°C until the shivering stopped. The total cooling time was 120 minutes. During the last 30 minutes of the cooling the second measurement of EE (EE_cold_) was performed.

### Measurement of Body Composition

Body composition was assessed by dual x-ray absorptiometry (Hologic Discovery W, Hologic, Marlborough, MA) at the Department of Radiology, University Hospital Basel, within 2 weeks of the respective study visit.

### Measurement of Body Core Temperature and Skin Temperature

We measured core body temperature by infrared tympanometry (Braun, ThermoScan PRO 6000, Marlborough, MA) directly after each measurement of EE. The skin temperature was measured continuously at 11 defined body locations by wireless iButtons (Maxim Integrated, San Jose, CA) (18): 7 central locations (supraclavicular region [left and right], parasternal at the level of the second intercostal space [left and right], umbilicus, mid-thigh [left and right]), and 4 peripheral locations (middle of the lower arm palmar side, finger tip of the third finger of the nondominant hand, middle of the lower left leg, back of the left foot). An additional sensor logged the ambient temperature at the study location.

### Meteorological Data

Outdoor temperatures were recorded by the Institute for Meteorology, Climatology and Remote Sensing at the University of Basel at an urban meteorological station in close vicinity of the University Hospital. Daily mean, maximum, and minimum temperatures were provided for all days during the study period. Mean daily temperatures were averaged over a period of 7 days prior to the respective study visit using a sliding average function.

### Statistical Analysis

Data were analyzed using R Version 4.04 (19) and GraphPad Prism Version 9.0 (GraphPad, La Jolla, CA). Continuous data are given as mean ± SD unless stated otherwise. Pairwise comparisons were performed with paired t-tests. Repeated measures data were analyzed with mixed-effects models in GraphPad Prism. More complex mixed-effects models were generated using the package “nmle” for R, Version 3.1-152 (20). Continuous variables included into the models were scaled by Z-scaling. A *P* value below .05 was considered statistically significant.

## Results

### Baseline Patient Data and Hormone Levels

We screened all patients attending our outpatient thyroid clinic for eligibility and recruited 19 patients for the study. At screening, all patients were hyperthyroid with a TSH level below 0.2 mU/L and fT4 at or above 25 pmol/L or fT3 at or above 8 pmol/L. However in 1 female patient with Graves’ disease there was a time lag of 18 days between the screening and the first study visit. At this timepoint the patient had levels of fT4 in the hypothyroid range. Therefore we excluded this patient from the analysis.

The analysis was performed in 18 patients (14 female, 4 male), 15 of the patients suffered from Graves’ disease, 2 had subacute thyroiditis, and 1 had iatrogenic hyperthyroidism due to overtreatment with levothyroxine (T4). The diagnosis of subacute thyroiditis was based on localized pain, negative antibodies, and matching ultrasound findings. In these 2 patients hyperthyroidism resolved without the use of antithyroid drugs.

Patients were on average 45.7 (range 20-70) years old when they were enrolled into the study. The mean duration between the first and the second visit were 122 ± 50 days and the duration between the first visit and the last visit were 240 ± 55 days. Clinical characteristics and laboratory data are given in Table 1.

**Table 1.** Clinical characteristics (mean ± SD)

	Baseline (n = 18, cold exposure n = 14)	Euthyroid (n = 16, cold exposure n = 12)	3 mo Euthyroid (n = 12, cold exposure n = 7)
Age (in years)	45.7 ± 15.1		
Sex	4 male/14 female	4 male/12 female	3 male/9 female
Cause of hyperthyroidism	15 Graves’ disease 2 subacute thyroiditis 1 overtreatment (levothyroxine 150 µg)	13 Graves’ disease 2 subacute thyroiditis 1 post overtreatment	9 Graves’ disease 2 subacute thyroiditis 1 post overtreatment
Weight (kg)	67.5 ± 18.3	70.9 ± 19.1	73.5 ± 22.1
Height (cm)	167.5 ± 9.4		
BMI (kg/m^2^)	23.7 ± 4.5	24.8 ± 4.7	25.4 ± 5.3
Lean mass (kg)	40.8 ± 11.4	43.7 ± 12.1	43.9 ± 13.0
Fat mass (kg)	22.8 ± 8.8	22.5 ± 8.9	24.9 ± 10.1
REE (kcal/24 h)	1654 ± 301	1443 ± 326	1433 ± 336
EE warm (kcal/24 h)	1667 ± 299	1519 ± 339	1582 ± 351
EE cold (kcal/24 h)	1722 ± 200	1569 ± 269	1583 ± 273
TSH (mU/L)	0.0076 ± 0.0062	1.98 ± 1.9	1.75 ± 1.3
free T4 (pmol/L)	36.3 ± 18.1	15.0 ± 3.9	16.6 ± 3.7
free T3 (pmol/L)	11.2 ± 5.1	4.3 ± 1.3	4.4 ± 0.6

Abbreviations: BMI, body mass index; EE, energy expenditure; T3, triiodothyronine; T4, thyroxine; TSH, thyroid-stimulating hormone.

### Thyroid Hormone Levels and Resting Energy Expenditure

**Figure 1.**
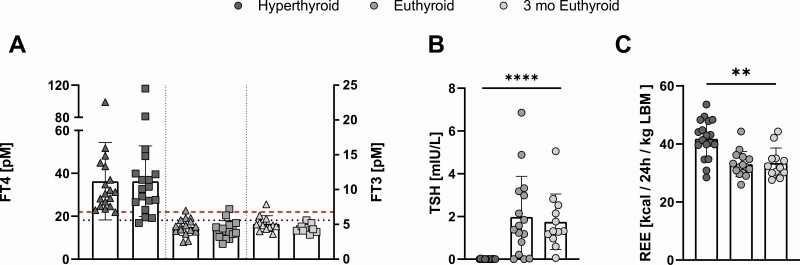
Thyroid hormone levels during hyperthyroid state, after restoration of euthyroidism and 3 months after restoration of euthyroidism. Effect of TH status on free T4 *P* = .0006, *P* = 0.0014 for pair-wise comparison hyperthyroid vs euthyroid and *P* = .020 for hyperthyroid vs 3 months euthyroid. Effect of TH status on free T3 *P* = .0002. Pairwise comparison hyperthyroid vs euthyroid *P* = .0001 and hyperthyroid vs 3 months euthyroid *P* = .0014 (A). TSH was suppressed during hyperthyroidism and normalized subsequently, *P* < .0001 (mixed-effects model) (B). REE (corrected to lean body mass) was elevated in hyperthyroidism and decreased significantly during course of the study, *P* < .0001 (mixed-effects model) (C).

TH levels decreased significantly during the course of the study (*P* = .0003 for fT4 and *P* = .0001 for fT3, Fig. 1A). At the initial visit, mean levels of fT4 were 35.8 ± 18.5 pM. At the second visit, after patients had reached the euthyroid hormone range, fT4 was 15.0 ± 3.9 pM (*P* = .0006) and remained approximately stable 3 months later (16.6 ± 3.7 pM, *P* = .015). Mean levels of fT3 decreased from 10.9 ± 5.3 pM (hyperthyroid) to 4.2 ± 1.3 pM (euthyroid) *P* = .0001 and 4.4 ± 0.6 pM (3 months euthyroid, *P* = .0014). TSH was suppressed during hyperthyroidism and normalized subsequently (Fig. 1B, *P* < .0001).

In parallel with the reduction of TH levels, REE decreased from 1654 ± 301 kcal/24 hours in the hyperthyroid state to a mean of 1443 ± 326 kcal/24 hours (–13%, *P* = .0034 vs hyperthyroid) in the euthyroid state and 3 months later to 1433 ± 336 (–13.4% vs hyperthyroid, *P* = .01; *P* = .0007 for overall effect of TH status). We also normalized REE to lean body mass: mean normalized REE was 42 ± 6.7 kcal/kg/24 hours during the hyperthyroid state, 33 ± 4.4 kcal/kg/24 hours once euthyroid TH levels were reached (–21%, *P* < .0001 vs hyperthyroid), and 33 ± 5.2 kcal/kg/24 hours after 3 months of euthyroid hormone values (–21%, *P* = .0022 vs hyperthyroid; global effect of TH level *P* < .0001, see also Fig. 1C).

We analyzed the effect of TH levels on EE with mixed-effect models with the individual participant as random-effect variable. Models which contained fT4 or fT3 as fixed effects, respectively, showed the well-known significant effect of TH on REE (*P* = .0003 for fT4 and *P* = .0001 for fT3). However, within these simple models fT4 and fT3 explained only 11% or 13% of REE’s variance (marginal R^2^ = 0.11 for fT4 and marginal R^2^ = 0.13 for fT3).

In order to improve the predictive value of the model we added lean body mass, age, and sex as fixed effect variables to the statistical models with fT4 or fT3, respectively. The model parameters were all significant and the marginal R^2^ improved to 0.74 and 0.77 (Table 2).

**Table 2.** Mixed effects models for influence of thyroid hormone on REE

	Free T4					Free T3				
Random effects	Formula: ~1 | Subject_ID					Formula: ~1 | Subject_ID				
	(Intercept)		Residual			(Intercept)		Residual		
SD	0.108		0.501			0.000		0.478		
Fixed effects	REE ~ free T4 + Lean Mass + Age + Sex.					REE ~ free T3 + Lean Mass + Age + Sex.				
	Value	SE	DF	t	*P*	Value	SE	DF	t	P
(Intercept)	–0.061	0.119	25	–0.51	.61	–0.083	0.107	25	–0.78	.44
Thyroid hormone (free T4 or free T3)	0.389	0.083	25	4.68	.0001	0.433	0.079	25	5.49	<.0001
Lean mass	0.722	0.152	25	4.75	.0001	0.706	0.136	25	5.19	<.0001
Age	–0.156	0.086	15	–1.82	.089	–0.100	0.078	15	–1.28	.22
Sex (M vs F)	0.259	0.342	15	0.75	.46	0.342	0.308	15	1.11	.28
R^2^	Conditional R^2^= 0.75, marginal R^2^ = 0.74					Conditional R^2^= 0.77, marginal R^2^ = 0.77				

All continuous variables were z-scaled prior to the analysis.

Moreover, we determined the respiratory quotient (RQ), a marker of metabolic fuel selection. RQ was 0.750 during hyperthyroidism, 0.745 after euthyroid TH had been attained and 0.748 after 3 months of euthyroid state (*P* = .96 for effect of TH status, see also Figure S1A (21)).

### Core Body Temperature and Skin Temperature

**Figure 2.**
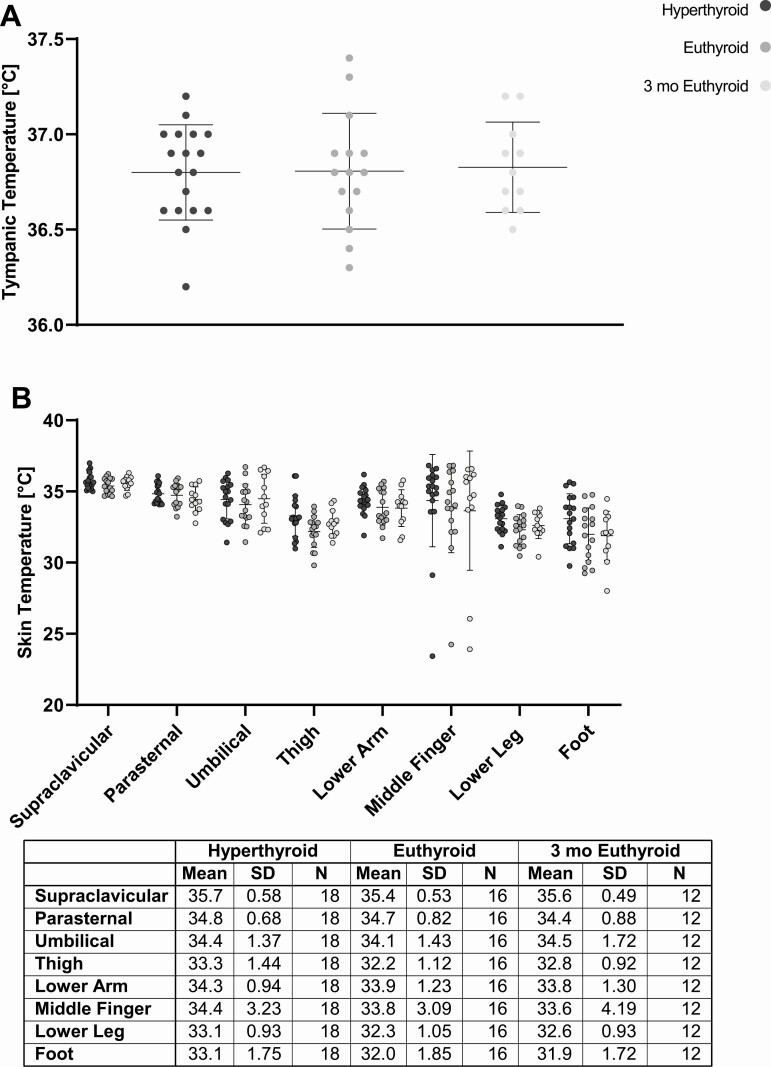
(A) Tympanic temperature as a surrogate of core body temperature did not change from the hyperthyroid to the euthyroid state. (B) Skin surface temperatures measured at 8 specified locations were higher in the hyperthyroid than in the euthyroid state, *P* < .0001 for effect of TH status (mixed-effects model).

Hyperthyroidism is known to increase core body temperature in rodents (22) and to cause hyperpyrexia in patients with thyroid storm (23). We therefore recorded tympanic temperature at each study visit. The mean tympanic temperature, as a surrogate marker of core body temperature, did not change from the hyperthyroid to the euthyroid state and was 36.8 ± 0.25°C (hyperthyroid), 36.8 ± 0.30 (euthyroid), and 36.8 ± 0.24 (3 months euthyroid, Fig. 2A), *P* = .95 for effect of TH status. We also measured skin surface temperature in 8 predefined regions (Fig. 2B). Over all, skin temperature was higher during hyperthyroidism than after normalization of TH levels, *P* < .0001 for effect of TH status (mixed-effects model).

### Resting Energy Expenditure at Cold Temperatures

**Figure 3.**
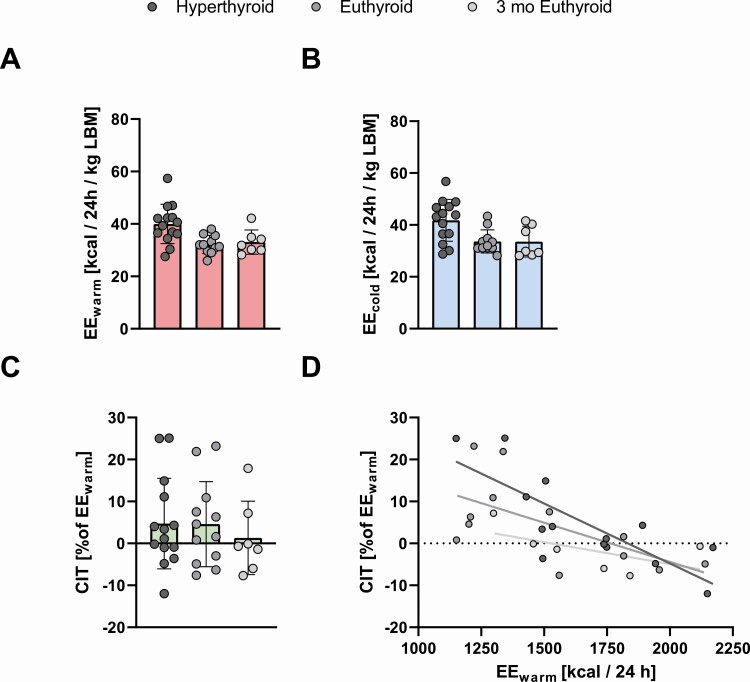
In a subset of participants we measured EE during warm conditions (EE_warm_) (A) and after a mild cold stimulus of 2 hours’ duration (EE_cold_) (B). EE values are corrected to lean-body mass. The increase in EE in response to cold, cold-induced thermogenesis (CIT), did not change over the course of the study (C) (*P* = .95, mixed-effects model). Further analysis revealed that CIT was inversely correlated to EE_warm_ especially during the hyperthyroid state (hyperthyroid, R^2^ = 0.63, *P* = 0.0007; euthyroid, R^2^=0.39, *P* = .031; 3 months’ euthyroid, R^2^ = 0.35, *P* = .22) (D).

Previously, hypothyroidism has been shown to decrease BAT activity and CIT (14, 15). A subgroup of 14 patients participated in additional visits in which mild cold exposure was used to assess CIT. We measured EE before (Fig. 3A) and after a mild cold stimulus of 120-minute duration (Fig. 3B) in the hyperthyroid and euthyroid state. As body composition changes during the treatment of hyperthyroidism, we normalized EE for lean body mass as determined by dual x-ray absorptiometry scan at the respective visit. During hyperthyroidism mean EE_warm_ was 40 ± 7.5 kcal/24 hour/kg lean body mass (LBM) and mean EE_cold_ was 42 ± 8.1 kcal/24 hour/kg LBM (*P* = 0.13 vs EE_warm_), mean relative CIT was 4.7 ± 11 % of EE_warm_. After TH levels had normalized, EE_warm_ was 32 ± 3.5 kcal/24 hour/kg LBM and EE_cold_ was 34 ± 4.5 kcal/24 hour/kg LBM (*P* = .18 vs EE_warm_) and CIT was 4.6 ± 10 % of EE_warm_. Three months later mean EE_warm_ was 33 ± 4.6 kcal/24 hour/kg LBM and mean EE_cold_ was 34 ± 5.9 kcal/24 hour/kg LBM (*P* = .66 vs EE_warm_), CIT was 1.3 ± 8.7% of EE_warm_. The change in CIT across the 3 study visits was not significant (*P* = .96) (Fig. 3C). RQ was not significantly affected by thyroid hormone state (*P* = .19) or cold exposure (*P* = .12, Figure S1B and 1C (21)).

In order to investigate the effects of TH levels on CIT we employed mixed-effects models with the individual subject as random effect and fT4 or fT3, respectively, as fixed effects. Consistent with the observation that CIT did not change across the 3 study visits, neither fT4 nor fT3 was associated with CIT (*P* = .54 and *P* = .59, respectively). We speculated that the elevated levels of EE during warm conditions (EE_warm_) due to hyperthyroidism negatively affected CIT. Indeed, EE_warm_ was inversely associated with CIT (*P* = .0003, marginal R^2^ = 0.46). We therefore created a mixed-effects model containing both REE and fT4 or fT3. As the seasonality of outdoor temperature is also known to significantly influence CIT (24), we also added the average maximum temperature during the week preceding the visit to the model. The fixed effects in these models explained 62% and 63% of the variance in CIT (Table 3). EE_warm_ was the strongest predictor of CIT (*P* = .0001) and was negatively associated. fT4 (*P* = .077) and fT3 (*P* = .029) were positively associated with CIT. Simple linear regression of EE_warm_ vs relative CIT (Fig. 3D) reveals that the inverse relation is especially pronounced during hyperthyroidism (R^2^ = 0.63, *P* = .0007, n = 14), but persists in the euthyroid state (R^2^ = 0.39, *P* = .031, n = 12). The coefficient of determination was similar after 3 months of stable euthyroid state (R^2^ =0.35) but the association was not significant, probably due to drop out of participants (*P* = .22, n = 7).

**Table 3.** Mixed effects model for influence of thyroid hormone on relative CIT

	Free T4					Free T3				
Random effects	Formula: ~1 | Subject_ID					Formula: ~1 | Subject_ID				
	(Intercept)		Residual			(Intercept)			Residual	
SD	0.335		0.491			0.323			0.486	
Fixed effects	Relative CIT ~ EE_warm_ + free T4 + TempMax7d + EE_warm_ : free T4					Relative CIT ~ EE_warm_ + free T3 + TempMax7d + EE_warm_ : Free T3				
	Value	SE	DF	t	*P*	Value	SE	DF	t	*P*
(Intercept)	0.051	0.137	15	0.372	.72	0.0583	0.134	15	0.434	.67
EE_warm_	–0.702	0.134	15	–5.24	.0001	–0.709	0.132	15	–5.37	.0001
Thyroid hormone (free T4 or free T3)	0.197	0.104	15	1.90	.077	0.253	0.105	15	2.41	.029
TempMax7d	–0.211	0.108	15	-1.95	.070	-0.214	0.105	15	–2.04	.060
Interaction EE_warm_:Thyroid hormone	–0.233	0.119	15	-1.97	.068	-0.256	0.104	15	–2.45	.027
R^2^	Conditional R2 = 0.74, marginal R2 = 0.62					Conditional R2 = 0.75, marginal R2 = 0.63				

All continuous variables were z-scaled prior to the analysis.

### Response of Skin Temperature to Cold Exposure and Influence of Thyroid Hormone State

**Figure 4.**
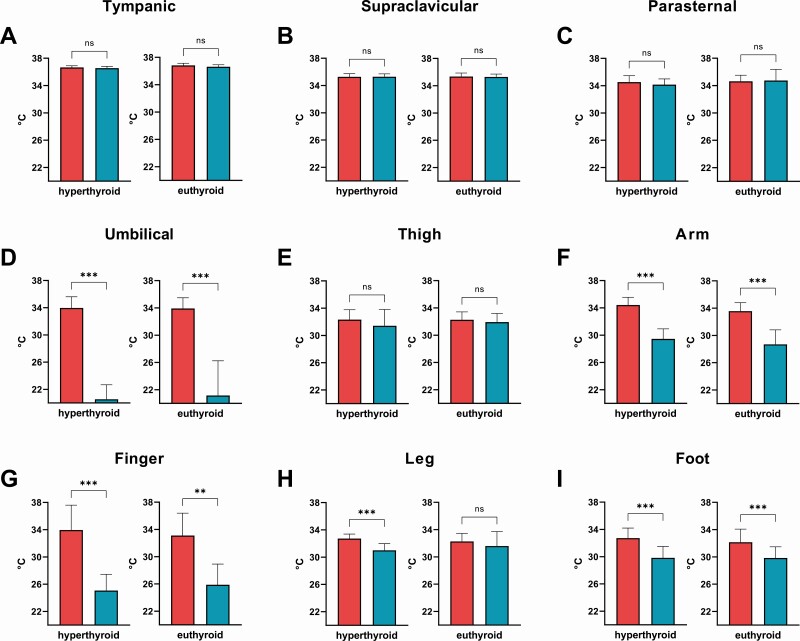
Tympanic (A) and skin temperatures (B-I) during the hyperthyroid state vs the euthyroid state at warm ambient temperature (red) and after mild cold exposure (blue). Cold exposure had a significant impact on skin temperature at the umbilicus (where the cooling device was placed) as well on the skin temperature of the lower arm, finger, lower leg, and foot. TH status did not significantly impact the response to cold (*P* for interaction > .05). **P < .01; ***P < .001; ns, *P* ≥ .05.

**Figure 5.**
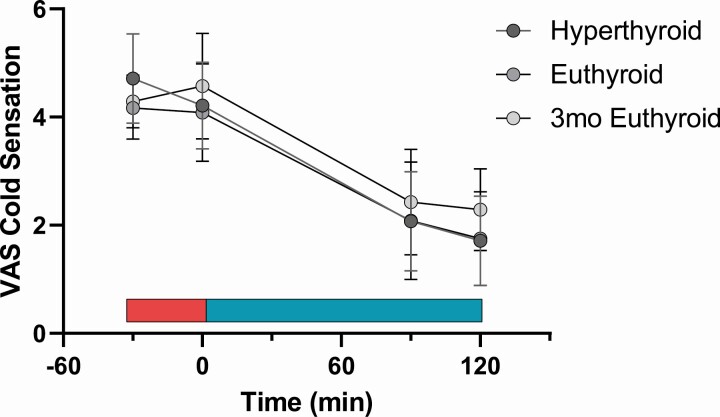
Subjective cold sensation on a visual analog scale (VAS) during the course of the measurement of CIT. Red bar: warm phase; blue bar: cooling phase. 7 = hot, 6 = warm, 5 = slightly warm, 4 = neutral, 3 = slightly cool, 2 = cold, 1 = very cold. No significant difference due to TH status was observed.

Cold exposure leads to reactive vasoconstriction of cutaneous vessels in order to reduce heat loss and thus lower skin temperature. Therefore, we measured tympanic and skin temperature at 8 locations before and after the cold exposure (Fig. 4A-4I). Cold exposure had a significant impact on skin temperature at the umbilicus (where the cooling device was placed) as well on the skin temperature of the lower arm, finger, lower leg, and foot. We tested whether the TH status (hyperthyroid vs euthyroid) had a significant impact on the temperature reaction in response to cold by using a mixed-effects model for the respective location. TH status did not significantly impact the response to cold (*P* for interaction >.05). The change in temperature at the respective location is given in Figure S2 (21). Importantly, the sensation of cold by study participants, as assessed by VAS was not influenced by the TH status (Fig. 5).

### BMI and Body Composition

Weight loss is a common symptom of hyperthyroidism. However, many patients experience weight gain exceeding their predisease weight when hyperthyroidism is treated successfully. We thus monitored patients’ weight and body composition during the course of the study.

**Figure 6.**
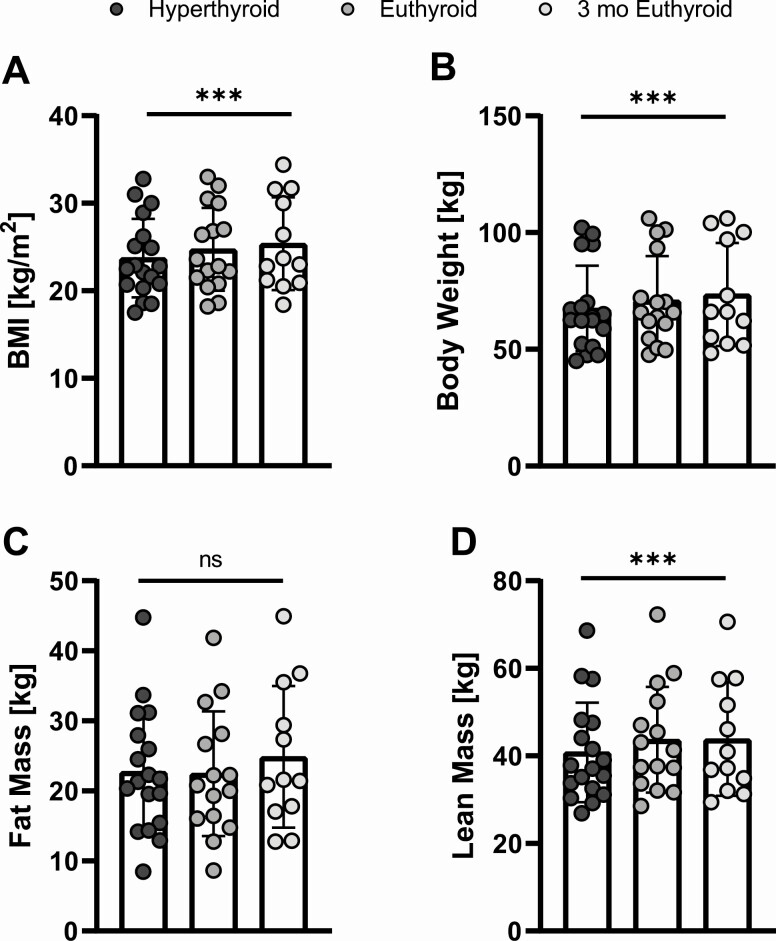
Body weight and body composition during the course of the study. (A) Body mass index (BMI) in kg/m^2^. (B) Total body weight in kg. (C) Fat mass in kg. (D) Lean mass in kg. ***P < .001 (mixed-effects model). ns, *P* ≥ .05.

The mean BMI in the hyperthyroid state was 23.7 ± 4.5 kg/m². In the euthyroid state the mean BMI increased to 24.8 ± 4.7 kg/m² (*P* = .001) and further to 25.4 ± 5.3 kg/m² after 3 months of normal TH levels (*P* < .001) (Fig. 6A). Total body weight increased significantly from 67.5 ± 18.3 kg (hyperthyroid) to 70.9 ± 19.1 kg (euthyroid) and 73.5 ± 22.1 kg (3 months euthyroid) (Fig. 6B). Of note, the mean fat mass in the hyperthyroid state was 22.8 ± 8.8 kg and remained almost unchanged after normalization of TH levels 22.5 kg ± 8.9 (*P* = .82, Fig. 6C). However, it increased significantly to 24.9 ± 10.1 kg after 3 months in the euthyroid state (*P* = .025 compared with the hyperthyroid state and *P* = .0018 compared with the recently euthyroid state). The mean lean body mass was 41 kg ± 11 kg in the hyperthyroid state and increased to a mean of 44 ± 12 kg in the euthyroid state significantly (*P* < .0001) and stabilized at 44 ± 13 kg after 3 months in the euthyroid state (*P* = .0001, Fig. 6D).

### Influence of Thyroid Hormone Excess and Energy Expenditure on Weight Change

We speculated that the degree of TH excess might influence weight regain after treatment of hyperthyroidism. We compared the increase in body weight, lean mass, and fat mass with levels of TH by Pearson correlation. This analysis was also performed with REE and CIT. As we performed multiple testing, we corrected the level of significance by Bonferroni correction (α* = 0.002). Using these criteria we did not observe a significant effect of TH level, REE, or CIT on the weight change (Table 4).

**Table 4.** Correlation of thyroid hormone levels and energy expenditure to body weight, lean mass and fat mass

Baseline		ΔBody weight (%)		ΔLean mass (%)		ΔFat mass (%)	
Hyperthyroid		Euthyroid	3 mo euthyroid	Euthyroid	3 mo euthyroid	Euthyroid	3 mo euthyroid
Free T4	*P*	.70	.072	.30	.069	.15	.30
	r	–0.11	0.54	0.29	0.54	–0.39	–0.33
	R^2^	0.01	0.29	0.083	0.29	0.15	0.11
Free T3	*P*	.98	.046	0.32	.11	.57	.57
	r	–0.01	0.58	0.28	0.48	–0.16	-0.18
	R^2^	0	0.34	0.076	0.23	0.025	0.032
REE	*P*	.93	.64	.07	.054	.86	.54
	r	–0.023	-0.15	-0.48	-0.57	0.05	0.19
	R^2^	0.0005	0.023	0.23	0.32	0.0025	0.038
CIT	*P*	.58	.39	.23	.14	.79	.72
	r	0.18	0.35	0.47	0.57	0.091	–0.15
	R^2^	0.032	0.12	0.14	0.33	0.0083	0.023

Pearson correlation.

## Discussion

In this study, we investigated the impact of hyperthyroidism on CIT in patients before and after treatment for primary hyperthyroidism. We hypothesized that overt hyperthyroidism in addition to its effect on REE also increases EE after mild cold exposure. Contrary to our hypothesis, CIT was not higher in the hyperthyroid state. Previously, CIT and BAT activity had been investigated in 6 patients with differentiated thyroid carcinoma who received TSH-suppressive doses of thyroxine. The increased TH levels did not lead to higher CIT or cold-induced BAT activity (25) which is in line with our findings.

CIT is the combined thermogenic response of BAT, skeletal muscle, and possibly other tissues to cold exposure (26). It is well established that TH is crucial for mitochondriogenesis and thermogenesis in BAT (12, 13, 27), and data from studies in humans indicate that hypothyroidism blunts the thermogenic response to cold (14, 15). Hyperthyroid levels of TH, on the other hand, have been shown to activate BAT in vitro (28), but the situation in vivo is much more complex. Hyperthyroid mice exhibit increased browning of otherwise white adipose tissue depots, so called “beige” adipose tissue, which is paralleled by a higher expression of UCP1 (29). Thyrotoxic treatment elevated REE at thermoneutrality both in wild-type and UCP1 knockout (KO) mice. The extremely high doses of thyroxine in this animal model, however, raised core body temperature at thermoneutrality and thus induced pyrexia. Both wild-type and UCP1 KO animals defended the increased core body temperature across a wide range of temperatures, but UCP1 KO mice failed to keep body core temperatures stable at temperatures around 4°C, supporting a role for both UCP1-dependent and -independent processes involved in thermogenesis during hyperthyroidism (22).

The elevated core body temperature is a hallmark of hyperthyroidism which has been induced by highly supraphysiologic doses of TH in animals (22, 30). In humans suffering from hyperthyroidism, elevated core body temperature is rare, occurring mainly in the setting of concomitant disease (31, 32). Hence, we did not observe elevated core body temperatures in the hyperthyroid compared with the euthyroid state. However, this does not preclude an increase of core body temperature above 37°C if the ambient temperature is considerably higher than the temperature in our study environment. Skin temperatures in the warm state were significantly higher during hyperthyroidism, indicating increased cutaneous heat loss as a consequence of elevated REE. TH signaling is crucial for the regulation of vascular tone and heat dissipation (33). Higher levels of TH might thus lead to improved insulating response to cold exposure and minimize the need for CIT. However, after mild cold exposure skin temperatures did not differ between the hyperthyroid and the euthyroid state, suggesting that the vasoconstrictory reactions to cold were comparable. In this context, it is of note that the subjects’ sensation of cold was not independent of the TH state.

In our study cohort, both fT4 and fT3 had a significant impact on REE. As elevated levels of fT4 and fT3 had such a pronounced influence on REE, we analyzed its effect on CIT. Indeed, the level of REE was inversely and significantly associated with CIT, demonstrating that higher levels of REE reduce the body’s need to increase EE in response to cold. This relation was especially apparent in the hyperthyroid state thus reiterating earlier findings from rodents: BAT activity in response to cold was blunted in thyroidectomized, hypothyroid rats and could be restored by replacing maintenance doses of TH. However, excessive amounts of TH did not increase BAT activity further (10). When we statistically corrected for the effect of REE on CIT by using mixed-effects modelling, fT4 and fT3 were positively associated with CIT albeit borderline significant. Taken together these findings indicate that the higher REE which is robustly induced by hyperthyroidism reduces the need for CIT and thus counteracts the permissive effects of TH on BAT thermogenesis.

Lower capacity of CIT has recently been shown to be associated with a higher propensity to weight gain underscoring a potential role of BAT and CIT in the prevention of obesity (34). Weight loss is common in hyperthyroidism, but a significant proportion of patients treated for hyperthyroidism quickly regains weight when returning to the euthyroid state, sometimes even exceeding predisease body weight (35, 36). We wondered whether weight regain was associated with changes in thermogenesis during hyperthyroidism. The increase in body weight could also be observed in our cohort, with a gain of almost 10%. It was mainly driven by lean body mass which has been observed previously (37). However, the weight change was neither related to excess REE during the hyperthyroid phase nor CIT.

Our conclusions are limited by the fact that we assessed global thermogenic response to cold exposure as measured by CIT. In humans, CIT is not solely facilitated by activation of BAT but caused skeletal muscle even in the absence of shivering (26, 38). We could not analyze the specific contributions of BAT or skeletal muscle to REE or CIT directly with ^18^F-fluorodeoxyglucose positron emission tomography/computed tomography. Previously, it was shown that under warm ambient conditions muscle metabolism is increased in hyperthyroidism and is the main contributor to the elevated REE but these studies did not expose patients to mild cold (17, 39). The supraclavicular skin temperature has been used as a surrogate parameter for BAT activity in humans (40) and has previously been shown to be reduced in hypothyroidism (15, 41). In this study supraclavicular skin temperature was higher during hyperthyroidism under warm conditions in line with increased BAT activity as previously described (17). However, it did not differ significantly between the hyperthyroid and euthyroid states after cold exposure. Our study population was very heterogeneous with a wide distribution of age, weight, and TH levels, which is often the case in clinical or translational studies. As we performed an observational study, we cannot directly prove causality. However, given the time course of TH levels and changes in EE causality is highly likely.

Our study is the first to systematically evaluate CIT and thermoregulation in patients with primary hyperthyroidism and reflects the average patient population presenting to an endocrine clinic. Further strengths of our study are a long follow-up in stable euthyroid state and statistical evaluation of environmental factors influencing CIT.

In conclusion, we show that CIT is not elevated in patients with hyperthyroidism. Our data indicate that this is likely a consequence of the high REE in these persons which makes additional thermogenesis unnecessary under conditions of mild cold exposure.

## Data Availability

Some or all datasets generated during and/or analyzed during the current study are not publicly available but are available from the corresponding author on reasonable request.

## Abbreviations

ATP: adenosine triphosphate
BAT: brown adipose tissue
BMI: body mass index
BMR: basal metabolic rate
cAMP: cyclic adenosine monophosphate
CIT: cold-induced thermogenesis
DIO2: deiodinase 2
EE: energy expenditure
fT3: free triiodothyronine
fT4: free thyroxine
HbA1c: glycated hemoglobin
KO: knockout
LBM: lean body mass;
PET: positron emission tomography
RAI: radioactive iodine
REE: resting energy expenditure
RQ: respiratory quotient
SNS: sympathetic nervous system
TH: thyroid hormone
TR: thyroid hormone receptor
TSH: thyroid-stimulating hormone
UCP1: uncoupling protein 1
VAS: visual analog scale
WAT: white adipose tissue

## Clinical Trials Information

ClinicalTrials.gov: NCT03379181 (registered December 20, 2017).

## Additional Information

Disclosures: The authors declare no conflict of interest.
